# Association between smoking and central sensitization pain: a web-based cross-sectional study

**DOI:** 10.1007/s00540-023-03302-4

**Published:** 2024-01-24

**Authors:** Satoko Chiba, Keiko Yamada, Aiko Kawai, Saeko Hamaoka, Hiroko Ikemiya, Atsuko Hara, Kenta Wakaizumi, Takahiro Tabuchi, Keisuke Yamaguchi, Izumi Kawagoe, Masako Iseki

**Affiliations:** 1 Department of Anesthesiology and Pain Medicine, Juntendo University School of Medicine, 2-1-1 Hongo, Bunkyo-Ku, Tokyo, 113-8421 Japan; 2 Department of Anesthesiology, Keio University School of Medicine, Tokyo, Japan; 3 Interdisciplinary Pain Center, Keio University Hospital, Tokyo, Japan; 4 Osaka International Cancer Institute and Cancer Control Center, Osaka, Japan

**Keywords:** Central sensitization syndrome, Chronic pain, Catastrophic thinking, Smoking

## Abstract

**Purpose:**

This study aimed to investigate whether smoking is an independent risk factor for central sensitization syndrome (CSS) in individuals with pain as measured by the Central Sensitization Inventory (CSI).

**Methods:**

In 2020, we conducted an Internet survey targeting 2000 ordinary residents of Japan (aged 20–69 years) who had pain symptoms from October to November 2020. A multiple regression analysis was performed on the association between smoking status (nonsmokers and current smokers; Brinkman index) and CSI values. Moreover, compared to nonsmokers, the relative risk (RR) of the CSI cut-off score of 40 points or higher among current smokers was calculated using a modified Poisson regression model. Covariates included age, sex, body mass index, marital status, equivalized income, exercise habits, history of hypertension, history of hyperlipidemia, history of diabetes, pain chronicity, and Pain Catastrophizing Scale score.

**Results:**

This study analyzed 1,822 individuals (1,041 men and 781 women). Among those experiencing pain, current smoking was associated with the increase in CSI values (*β* = 0.07). The Brinkman index was also significantly associated with the increase in CSI values (*β* = 0.06). Current smoking also increased the risk of being over the CSI cut-off score, with a relative risk (RR) of 1.29 (95% confidence intervals, 1.04–1.60). Younger age, being women, experiencing chronic pain, and higher pain catastrophizing thinking were also significantly associated with increased CSS severity, independent of smoking status.

**Conclusion:**

Smoking is an independent risk factor for CSS. This indicates that smoking may be an important factor in the management of central pain disorders.

**Supplementary Information:**

The online version contains supplementary material available at 10.1007/s00540-023-03302-4.

## Introduction

Smoking is an independent risk factor in the onset and progression of persistent pain [1, 2]. A previous study reported that smoking was associated with the impact of pain on work [3]. Both systemic inflammation due to smoking [4] and the effect of nicotine on the central nervous system [5] have been identified as mechanisms associated with the relationship between smoking and the severity of persistent pain. However, the details of the mechanisms responsible remain unknown.

Central sensitization (CS) is a phenomenon where sensory input experiences heightened stimulation within the central nervous system, resulting in amplified pain signals [6]. This involves magnified nerve signals inducing hyperalgesia [6], dysfunction in pain inhibition [7, 8], and altered brain activity [8]. Central Sensitization Syndrome (CSS) is a condition associated with CS, characterized by various symptoms, including pain, fatigue, sleep disturbances, anxiety, and depression [9]. The Central Sensitization Inventory (CSI) is a questionnaire for quantifying CSS, the validity of which has been verified in Japan [10].

Smoking is known to be a bidirectional risk factor for depression [11], and is thought to affect the central nervous system. Consequently, it is plausible that smoking could contribute to the development of CSS. Our hypothesis is that smokers are likely to develop central sensitization as a consequence of smoking.

This study aimed to test the above hypothesis by examining the association between smoking and CSS of pain as measured by CSI using data from an Internet survey on smoking and pain administered to the general population of Japan.

## Methods

### Survey participants

**Fig. 1:**
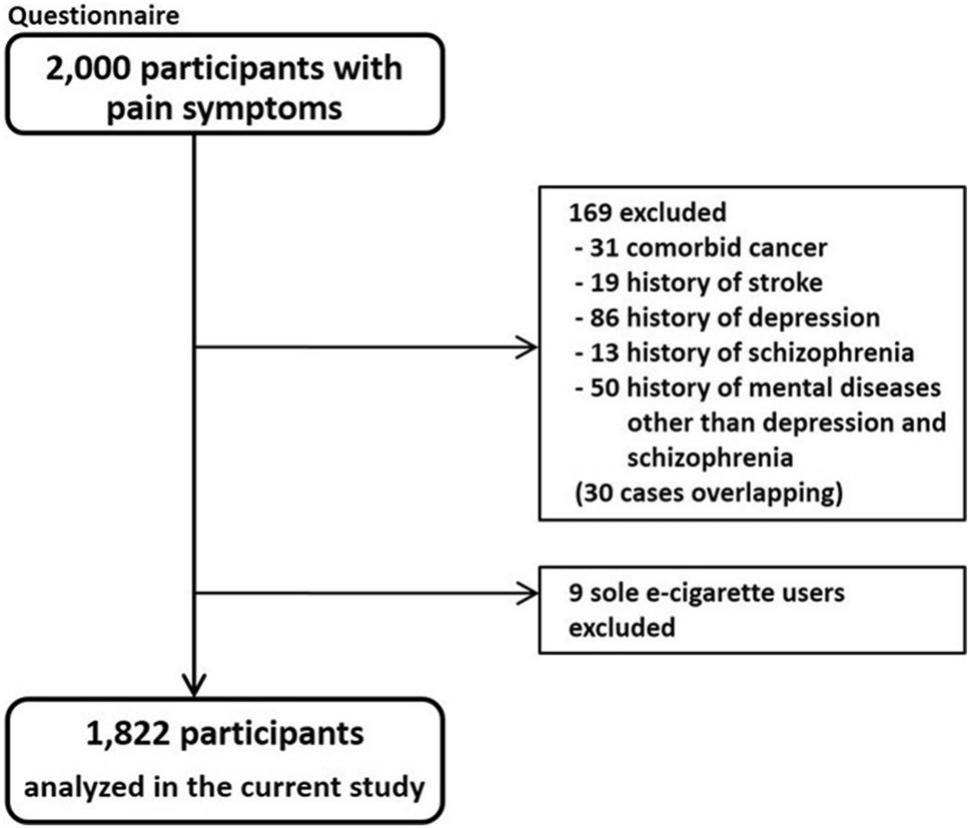
Participant enrollment process. Participants were recruited in 2020

This survey was conducted by a web research company (Rakuten Insight, Tokyo). The survey was administered to 2000 consenting panel monitors (aged, 20–69 years) in the general population who had experienced pain anywhere in the body from October to November 2020. This online system for this study only collected data from respondents who completed the questionnaire. The questionnaire took approximately 20–30 min to complete. We excluded 169 participants who were receiving treatment for comorbidities (31 with cancer, 19 with stroke, 86 with depression, 13 with schizophrenia, 50 with psychiatric disorders other than depression and schizophrenia, and 30 with multiple diseases listed above). Furthermore, because e-cigarettes in Japan do not contain nicotine (though heated tobacco products do) and this study seeks to test a hypothesis about the effects of nicotine in cigarettes on the central nervous system, the nine e-cigarette users were excluded for a total of 1822 participants (Fig. 1). Based on a previous epidemiological study that examined the relationship between smoking and pain among 1189 Japanese workers [3], we established a sample size of approximately double for this study.

### Main survey items

#### Basic characteristics

Age, sex, marital status, and equivalized income were examined. Marital status was divided into married (including remarriage), common-law marriage, unmarried, separated or divorced (no remarriage), and bereaved. Equivalized income was calculated by dividing annual household income by the square root of the number of family members. Poverty was defined with reference to the 2018 poverty line [12] as an equivalized income of less than 1.27 million yen.

#### Lifestyle and medical information

The survey collected information on obesity, exercise habits, and comorbidities. Body mass index (BMI) was calculated by body weight in kg/(height in m)^2^; those over 25 were defined as overweight. We investigated whether respondents had regular exercise habits (exercise up to a light sweat for 30 min or more at a time, for 2 days or more a week, and for 1 year or more; yes or no).

Concerning comorbidities, we surveyed whether respondents were receiving treatment for hypertension, hyperlipidemia, or diabetes.

#### Duration of pain

Duration of pain was classified into: less than 1 month; 1 to 3 months; 3 to 6 months; 6 months to 1 year; and 1 year or longer. Chronic pain was defined using the ICD-11 as pain that persists for more than 3 months [13].

#### Catastrophic thinking

Catastrophic thinking is defined as an exaggerated negative “mental set” brought to bear during painful experiences [14], and this abnormal perception of pain may be involved in cases of intractable pain. The Pain Catastrophizing Scale (PCS) is a self-report questionnaire that assesses catastrophic thinking related to pain. It consists of 13 items, with each item rated on a five-point Likert-type scale (0 = never; 4 = always). The possible score range is from 0 to 52, with higher scores indicating more intense catastrophic thinking. The PCS comprises three subscales: 'ruminating', 'helplessness', and 'magnification' [15]. There have been reports of a correlation between smoking and the PCS [16]. Since there was a possibility that the PCS would be a confounding factor for this study, we examined the participants' PCS scores and adjusted for it in the statistical analysis. In this survey, we used the Japanese version of the PCS, which has been validated for reliability and validity previously [17].

#### Smoking status

The survey divided smoking status into the following four categories: daily, occasionally, stopped smoking, and never smoked. It also asked respondents the age at which they began smoking and the number of cigarettes smoked per day. The Brinkman index is calculated by multiplying the number of cigarettes smoked per day by the number of years of smoking. If the number of years spent smoking was less than 1, the Brinkman index was calculated using 0.5 as the number of years of smoking.

#### Central sensitization symptoms

Central Sensitization Symptoms were assessed using Part A of the CSI, a self-administered questionnaire. Part A of the CSI comprehensively evaluates subjective symptoms common to Central Sensitization Symptoms, such as pain, fatigue, sleep disorders, anxiety, depression, and cognitive impairment [10]. CSI-A consists of 25 items, and the frequency of subjective symptoms is evaluated on a five-point Likert-type scale of 0 to 4 (0 = never; 4 = always), with a total score of 0 to 100 points and a cut-off value of 40 [10, 18]. The distribution of CSI scores is shown in Supplementary Fig. 1.

#### Time discount rate coefficient

Continuous intake of nicotine induces desensitization of nicotine receptors, resulting in decreased function of the reward dopamine system, in which the reward system cannot be activated unless more nicotine is ingested [19]. However, it has also been reported that patients with chronic pain have reduced activity signals in the reward system regions of the brain, including the nucleus accumbens [20]. The time discount rate is involved in decision-making associated with the reward prediction function of the reward system (the process of deciding whether to prioritize immediate rewards or future rewards that are valuable to oneself) [21]. Since there was a possibility that the reward system would be a confounding factor for this study, the survey investigated the participants’ time discount rate coefficient. In response to the question, "For a gratuity of XXX yen paid today, would you feel that a gratuity of ¥YYY would be of equal value if it were paid ZZZ days later?", the survey instructed respondents to enter a numerical value into the "ZZZ" portion of the question. The survey included 10 items of this type, using somewhat different amounts each time. The functional form of the time discount coefficient uses a hyperbolic model of *V*(*D*) = *V*(0)/(1 + κ*D*) [*V*(*D*): delayed reward (XXX), *V*(0): immediate reward (YYY), *D*: number of days (ZZZ), κ: time discount coefficient], and when plotting the hyperbola for each individual, the coefficient κ was calculated.

### Statistical analysis

The explanatory variables were divided into two groups: a smoking group and a non-smoker group. Multiple regression analysis was performed using the CSI value as a continuous variable, ranging from 0 to 100 points, as the objective variable. Furthermore, these analyses were performed again after changing the smoking status to the Brinkman index.

A modified Poisson regression model was used to compare the relative risk (RR) and 95% confidence intervals (CIs) of exceeding the CSI cut-off score among smokers relative to nonsmokers. We used Statistical Analysis Software (SAS; SAS Institute Inc., Cary, NC, USA) version 9.4 and the REPEATED statement of SAS PROC GENMOD to estimate the sandwich error using the modified Poisson regression model.

In these analyses, Model 1 was adjusted for age and sex. Model 2 was further adjusted for BMI (quintile), marital status, equivalized income (quintile), regular exercise (yes/no), history of hypertension, history of hyperlipidemia, history of diabetes mellitus, and pain chronicity (defined as pain duration of 3 months or more). Model 3 included additional adjustments for the PCS score.

We excluded 120 participants with history of mental illnesses (such as depression, schizophrenia, and other psychiatric disorders) in our analysis to distinguish between major mental illnesses and central sensitivity syndromes (CSS). However, acknowledging the potential overlap between mental illnesses and CSS, we aimed to prevent misclassification. In the sensitivity analysis, we included these 120 participants, who were previously excluded from the main analysis due to their history of mental illnesses, which was considered a confounding factor. We re-ran the multiple regression analysis incorporating this group (*n* = 1942). In the sensitivity analysis, adjustments were made in Model 3, and further adjustments for the history of depression, history of schizophrenia, and history of other mental diseases were incorporated in Model 4.

SAS 9.4 was used for all the above data analysis, and statistical significance was set at *p* < 0.05 for both models.

### Ethical concerns

When administering the Internet survey, participants were presented with an explanatory letter outlining the survey, stating that the data would not be used for any other purpose, that their personal information would be protected through anonymization, and that they would not be disadvantaged if they refused to cooperate in the survey. Participants agreed to participate in the research by clicking the consent button and proceeding to the questionnaire response screen. Participants were incentivized with points that could be used for Internet shopping or converted to cash. The points awarded for survey cooperation are uniformly determined by the internet survey company, in accordance with established rules. However, researchers are not informed of the specific monetary value of this point system. This study was approved by the Ethics Committee of Juntendo University School of Medicine (approval number: 2020173, approval date: October 15, 2020).

## Results

The participants’ backgrounds are shown in Table 1 and Supplementary Table 1. The smoking rate was 22.6%. Compared to the nonsmoking group, the smoking group included fewer women and more participants who were overweight, had diabetic comorbidities, experienced chronic pain, and had higher PCS and CSI scores. There was no statistically significant difference in time discounting between the nonsmoking and smoking groups.

**Table 1:** Mean values and proportions for demographic factors (*n* = 1822)

	Total		Non-smoker		Smoker		*P* value
	*n* = 1822		*n* = 1411		*n* = 411		
	Mean	SD	Mean	SD	Mean	SD	
Age, years	45.6	13.4	45.5	13.7	46.1	12.4	0.43

	*n*	(%)	*n*	(%)	*n*	(%)	
Women	781	42.9	687	48.7	94	22.9	< 0.001
BMI ≥ 25 kg/m^2^	395	21.7	287	20.3	108	26.3	0.01
Divorced or widowed	158	8.7	115	8.2	43	10.5	0.14
Poverty	38	2.1	28	2.0	10	2.4	0.58
Lack of regular exercise	1257	69.0	979	69.4	278	67.6	0.50
History of hypertension	194	10.6	149	10.6	45	10.9	0.82
History of hyperlipidemia	113	6.2	91	6.4	22	5.4	0.42
History of diabetes mellitus	70	3.8	46	3.3	24	5.8	0.02
Pain duration ≥ 3 months	884	48.5	654	46.4	230	56.0	< 0.001
Primary site of pain							
Head	224	12.3	171	12.1	53	12.9	
Orofacial	108	5.9	80	5.7	28	6.8	
Throat	13	0.7	11	0.8	2	0.5	
Neck or shoulder	448	24.6	333	23.6	115	28.0	
Upper limb or hand	38	2.1	79	5.6	17	4.1	
Abdomen	133	7.3	115	8.2	18	4.4	
Back	40	2.2	28	2.0	12	2.9	
Lower back	441	24.2	340	24.1	101	24.6	
Inguinal or femoral	64	3.5	52	3.7	12	2.9	
Knee	131	7.2	106	7.5	25	6.1	
Leg or foot	108	5.9	84	6.0	24	5.8	
Genitals or anus	16	0.9	12	0.9	4	1.0	

	Mean	SD	Mean	SD	Mean	SD	
PCS score	18.6	11.6	18.2	11.6	19.6	11.7	0.03
CSI score	25.1	14.9	24.7	14.7	26.5	15.6	0.03
Time discounting	− 2.7	1.6	− 2.7	1.6	− 2.6	1.6	0.20
Never-smoker			1044	57.3	**–**	**–**	
Former smoker			367	20.1	**–**	**–**	
Number of cigarettes per day							
≥ 1 and < 10			**–**	**–**	109	26.5	
≥ 10 and < 20			**–**	**–**	123	29.9	
≥ 20 and < 40			**–**	**–**	138	33.6	
≥ 40					41	10.0	
Smoking duration (mean 25.0, SD 12.7)							
≥ 0 and < 1 year					6	1.5	
≥ 1 and < 10 years					61	14.8	
≥ 10 and < 20 years					72	17.5	
≥ 20 and < 30 years					109	26.5	
≥ 30 years					163	39.7	
Brinkman index							
Q1: 0.5–145					103	25.1	
Q2: 148–360					103	25.1	
Q3: 375–672					102	24.8	
Q4: 675–4312					103	25.1	

Never-smoker and ex-smoker were categorized as non-smoker

Poverty was defined as an equivalized income of less than 1.27 million Japanese yen

The number of cigarettes smoked per day multiplied by the number of years of smoking was defined as the Brinkman index

*SD* standard deviation, *BMI* body mass index, *PCS* Pain Catastrophizing Scale, *CSI* Central Sensitization Inventory, *Q* Quartile

Smoking between 20 and 40 cigarettes per day was the most common rate, accounting for 33.6% of the total. Smoking for more than 30 years was the most common smoking duration, accounting for 39.7% of the total.

Table 2 shows the results of a multiple regression analysis using the two smoking and nonsmoking groups as explanatory variables and the CSI value as the objective variable. Smoking was significantly associated with increased CSI values (Model 3: standardized regression coefficient (*β*) = 0.05 [95% confidence interval (CI) 0.01–0.09, *p* = 0.03]).

**Table 2:** Standardized regression coefficient (95% confidence intervals) for the Central Sensitization Inventory (CSI) score

	Model 1:			Model 2			Model 3		
	*β*	95% CI	*p* value	*β*	95% CI	*p* value	*β*	95% CI	*p* value
Smoker (ref. non-smoker)	0.08	0.04 to 0.13	< 0.001	0.07	0.02 to 0.12	0.003	0.05	0.01 to 0.09	0.03
Age: 10 year increment	− 0.19	− 0.23 to − 0.14	< 0.001	− 0.22	− 0.27 to − 0.17	< 0.001	− 0.17	− 0.22 to − 0.13	< 0.001
Women (ref. men)	0.13	0.09 to 0.18	< 0.001	0.14	0.09 to 0.19	< 0.001	0.10	0.06 to 0.14	< 0.001
Chronic pain	–	–	–	0.20	0.16 to 0.24	< 0.001	0.11	0.07 to 0.14	< 0.001
PCS	–	–	–	–	–	–	0.48	0.44 to 0.52	< 0.001

*β* standardized regression coefficient, CI; confidence interval, PCS; Pain Catastrophizing Scale, ref; reference

Model 1: Adjusted for age and sex

Model 2: Adjusted for age, sex, body mass index, marital status, equivalized income, regular exercise, history of hypertension, history of hyperlipidemia, history of diabetes mellitus, and chronic pain

Model 3: Adjusted for age, sex, body mass index, marital status, equivalized income, regular exercise, history of hypertension, history of hyperlipidemia, history of diabetes mellitus, chronic pain, and Pain Catastrophizing Scale score

*n* = 1822

Independent of smoking, CSI values decreased with age (Model 3: *β* = − 0.17 [95% CI − 0.22 to − 0.13, *p* <0.001]), increased among women (Model 3: *β* = 0.10 [95% CI 0.06–0.14, *p* <0.001]), increased among individuals with chronic pain (Model 3: *β* = 0.11 [95% CI 0.07–0.14, *p* <0.001]), and increased among individuals with higher PCS scores (Model 3: *β* = 0.48 [95% CI 0.44–0.52, *p* <0.001]).

Table 3 shows the results of a multiple regression analysis using the Brinkman index as the explanatory variable and the CSI value as the objective variable. An increased Brinkman index was significantly associated with higher CSI values. Independently of an increased Brinkman index, CSI values decreased with increasing age, increased among women, and were higher among individuals with chronic pain and those with higher PCS scores.

**Table 3:** Standardized regression coefficient (95% confidence intervals) for the Central Sensitization Inventory (CSI) score

	Model 3		
	*β*	95% CI	*p* value
Brinkman Index	0.06	0.02 to 0.10	0.004
Age: 10 year increment	− 0.18	− 0.23 to − 0.14	< 0.001
Women (ref. men)	0.10	0.06 to 0.14	< 0.001
Chronic pain	0.11	0.07 to 0.15	< 0.001
PCS	0.48	0.44 to 0.52	< 0.001

Model 3: Adjusted for age, sex, body mass index, marital status, equivalized income, regular exercise, history of hypertension, history of hyperlipidemia, history of diabetes mellitus, chronic pain, and Pain Catastrophizing Scale score

The number of cigarettes smoked per day multiplied by the number of years of smoking was defined as the Brinkman index

*n* = 1822

*β* standardized regression coefficient, *CI* confidence interval, *PCS* Pain Catastrophizing Scale

Table 4 shows the association between smoking and the cut-off score of the CSI. Smoking was significantly associated with exceeding the cut-off score of the CSI [Model 3: RR = 1.29 (95% CI 1.04–1.60), *p* = 0.03]. Independent of smoking, younger age, being a woman, having pain chronicity, and a higher PCS score were each associated with exceeding the cut-off score of the CSI.

**Table 4:** The association between smoking and the cut-off score of the Central Sensitization Inventory score

	*n*	Number of case	Model 3
			RR (95% CI)
Non-smoker	1411	219	1
Smoker	411	85	1.29 (1.04–1.60)*
Age: 10 year increment			0.89 (0.86–0.93)***
Women (ref. men)			1.37 (1.12–1.68)*
Chronic pain			1.06 (1.05–1.07)**
PCS			1.06 (1.05–1.07)***

Model 3: Adjusted for age, sex, body mass index, marital status, equivalized income, regular exercise, history of hypertension, history of hyperlipidemia, history of diabetes mellitus, chronic pain, and Pain Catastrophizing Scale score

*CI* confidence interval, *RR* relative risk, *PCS* Pain Catastrophizing Scale

^*^p < 0.05, **p < 0.01, ***p < 0.001

The sensitivity analysis, which included participants previously excluded from the main analysis due to their exclusive history of depression, schizophrenia, and other mental diseases revealed results similar to the main findings (see supporting information, Table S2).

## Discussion

Among those experiencing pain, these results suggest that smokers may present with more severe CSS as compared to nonsmokers. The higher the Brinkman index, the more severe the CSS. Younger age, female sex, pain chronicity, and higher pain catastrophic thinking also significantly explained CSS severity independently of smoking. There was no difference in the time discount rate coefficient between nonsmokers and smokers in this study.

In this survey, CSS, a neurological change in the brain, was measured using CSI. In the previous studies, neurological changes in the brains of CSS patients have been investigated using brain imaging studies and Quantitative Sensory Testing (QST). QST is a method for objectively measuring sensitivity to sensory stimuli, commonly employed in clinical and research settings to evaluate sensory perception and detect abnormalities associated with pain or sensory processing. These previous study have reported that patients with CSS have decreased volume of brain regions associated with pain processing in the cerebral gray matter (i.e., anterior cingulate cortex and prefrontal cortex), decreased functional connectivity of descending pain regulatory systems, increased activity of the pain matrix associated with central sensitization [22], and reported reductions in nociceptive reflex thresholds [23], it is likely that CSS reflects neurophysiological changes in the brain, and estimating the degree of central sensitization with the CSI questionnaire is a reasonable approach. Therefore, we consider that CSI is a simple method that can be implemented in large surveys, such as this one, to estimate the degree of central sensitization in many people. However, additional evaluations based on imaging and physiological indices such as brain imaging and QST will make more objective CS evaluation possible in the future.

In the previous studies, higher pack-years of lifetime smoking have been associated with an increased risk of chronic pain, greater pain severity, and frequency [24]. This suggests a potential link between smoking and central sensitization, a process implicated in conditions like CSS. While smoking is known to be a risk factor for mental disorders [11], and a relationship between smoking and PCS scores has been suggested [16], our study found that the association between smoking and CSI scores remained robust even after accounting for mental disorders, PCS, and other factors. This suggests that smoking may directly contribute to central sensitization, leading to CSS, independently of mental status-related factors such as mental disorders or cognitive dysfunction.

These results are consistent with the previous studies that found age, the female sex, and pain catastrophic thinking also significantly explained the severity of CSS, independently of smoking. It has been reported that immune responses to neuroinflammation, which can lead to central pain processing dysfunction, differ by sex, with women more likely to develop central sensitization [25]. There are various theories about age and neuroinflammation [26–28], with one study finding that central sensitization accelerates with aging [29] and another finding that it decreases [30]. Although the significant finding that the association between smoking and CSI remained even after adjusting for age in this study is an important insight, further research is needed. Catastrophic thinking is defined as an individual's tendency to overestimate the disability resulting from current and future pain in their cognitive and emotional responses. Previous research has reported the possibility that negative expectations may alter brain circuits, including those involved in endogenous pain control [31, 32].

In general, the time discount rate is higher for smokers than for nonsmokers, as demonstrated by a systematic review and network analysis [33]. However, there was no difference in the time discount rate coefficient between nonsmokers and smokers in this study. We recruited individuals with pain symptoms, which can alter reward processing at both clinical and molecular levels [34, 35]. Therefore, the clear association between time discounting and smoking behavior may have disappeared in this study. In addition, nicotine use is associated with two inconsistent aspects of pain sensation: acute analgesic effects and hyperalgesia [36]. The complicated effects of smoking behavior on pain may potentially affect reward processing expressed by time discounting. To identify the mechanisms of smoking on the reward system in people with pain symptom, further large-scale survey will be required.

There are several epidemiological studies on the prevalence of chronic pain (defined as pain lasting longer than three months) in Japan. Reports indicated that 22.9% of individuals aged 20 years or older experienced chronic pain in 2009 [37] and 22.5% in 2010 [38]. These figures appear smaller than those found in the current study. However, it is important to note that these prevalences were specific to those experiencing moderate-to-severe pain, while our study investigated the entire spectrum of chronic pain. Our previous report in 2017 [39] revealed that the prevalence of chronic pain among workers aged 20–64 years was 42.7%, a finding consistent with the results of the current study. Consequently, we believe that the selection bias in the current study is not substantial.

This study has some limitations. The survey was conducted on the internet. The survey covered 2000 randomly selected participants according to population composition by sex, age, and prefecture, which we believe reflects the current status of smoking and pain among the Japanese population. However, since the target age group was set below 70 years of age, the results may have been different if the trends of smokers aged 70 and older in Japan—an aging society—were also examined. In addition, this was a cross-sectional survey, not a causal study. It is possible that participants smoke and have a high Brinkman index, because their CSS is severe. We believe that longitudinal studies evaluating the impact of these factors over time are needed to show that smoking contributes to the severity of CSS.

## Conclusion

Smoking is an independent risk factor for CSS. Furthermore, a higher Brinkman index is associated with the severity of CSS. Therefore, smoking is an important factor in addressing persistent pain among those experiencing pain.

### Supplementary Information

Below is the link to the electronic supplementary material.

Supplementary file1 (PDF 243 KB)

## Data Availability

The data used in this study are not available in a public repository because they contain personally identifiable or potentially sensitive patient information. Based on the regulations for ethical guidelines in Japan, the Research Ethics Committee of Juntendo University School of Medicine has imposed restrictions on the dissemination of the data collected in this study. All data enquiries should be addressed to the person responsible for data management, Dr. Satoko Chiba at the following e-mail address: sa-chiba@juntendo.ac.jp.
