# Supplementary material for: Association between smoking and central sensitization pain: a web-based cross-sectional study
Source: J Anesth. 2024 Jan 24;38(2):198–205. doi: 10.1007/s00540-023-03302-4 (PMC10954963; doi:10.1007/s00540-023-03302-4)
Supplement: Supplementary file 1 — Supplementary file1 (PDF 243 KB) [file 540_2023_3302_MOESM1_ESM.pdf]

Table S1. Participant characteristics (n=1,822)

	n	(%)
**Age, years**		
20–29	298	16.4
30–39	332	18.2
40–49	428	23.5
50–59	377	20.7
60–69	387	21.2
**Sex**		
Women	781	42.9
Men	1,041	57.1
**Body mass index, kg/m2**		
<18.5	191	10.5
18.5–24.9	1,236	67.8
25.0–29.9	311	17.1
≥30.0	84	4.6
**Educational attainment**		
Less than high school	26	1.4
High school	409	22.4
Technical college	35	1.9
Vocational school	225	12.3
Junior college	167	9.2
University	840	46.1
Graduate school	115	6.3
Other	5	0.3
**Marital status**		
Married or common law	1,059	58.1
Single	605	33.2
Divorced or separated	131	7.2
Widowed	27	1.5
**Employment status**		
Company executive	76	4.2
Owner of family operated business	147	8.1
Full-time employee	860	47.2
Dispatched employee	40	2.2
Contract employee	97	5.3
Part-time employee	203	11.1

Student	52	2.9
Full-time homemaker	193	10.6
Unemployed	154	8.5
**Equivalized income. million Japanese yen**		
Q1: –2.49	247	13.6
Q2: 2.50–3.49	266	14.6
Q3: 3.50–4.49	242	13.3
Q4: 4.50–5.78	269	14.8
Q5: 5.79–	241	13.2
Missing	566	31.1
**Regular exercise**		
Yes	565	31.0
No	1,257	69.0
**Sleep duration**		
<5 hours	196	10.8
≥5 hours, <6 hours	573	31.4
≥6 hours, <7 hours	668	36.7
≥7 hours, <8 hours	314	17.2
≥8 hours, <9 hours	58	3.2
≥9 hours	13	0.7
**History of hypertension**	194	10.6
**History of hyperlipidemia**	113	6.2
**History of diabetes mellitus**	70	3.8
**Medication**		
Over-the-counter analgesic	464	25.5
Prescribed analgesic	311	17.1
Hypnotic	57	3.1
Anxiolytic	19	1.0
Antidepressant	9	0.5
**Pain duration**		
<1 month	686	37.7
≥1 month, <3 months	252	13.8
≥3 month, <6 months	138	7.6
≥6 month, <1 year	94	5.2
≥1 year	652	35.8
	**Mean**	**SD**
**PCS score**	18.6	11.6
**CSI score**	25.1	14.9

**Pain intensity**		
Maximum	6.2	2.3
Average	4.8	2.0
Current	4.5	2.3

Abbreviations: Q, Quintile, SD; standard deviation, CSI; Central Sensitisation Inventory, PCS; Pain Catastrophizing Scale.

Table S2. Standardized regression coefficient (95% confidence intervals) for the Central Sensitisation Inventory (CSI) score

	**Model 3**			**Model 4**		
	**β**	**95% CI**	**p value**	**β**	**95% CI**	**p value**
**Smoker (ref. non smoker)**	0.06	0.02 to 0.10	0.03	0.05	0.01 to 0.08	0.03
**Age: 10 years increment**	-0.18	-0.23 to -0.14	<0.001	-0.17	-0.22 to -0.13	<0.001
**Women (ref. Men)**	0.10	0.06 to 0.14	<0.001	0.10	0.07 to 0.14	<0.001
**Chronic pain**	0.11	0.07 to 0.15	<0.001	0.10	0.07 to 0.14	<0.001
**PCS**	0.50	0.46 to 0.54	<0.001	0.48	0.44 to 0.51	<0.001
**History of depression**	-	-	-	0.13	0.10 to 0.17	<0.001
**History of schizophrenia**	-	-	-	0.03	-0.004 to 0.17	0.08
**History of other mental diseases**	-	-	-	0.07	0.03 to 0.11	<0.001

β; standardized regression coefficient, CI; confidence interval, PCS; Pain Catarsophizing Scale, ref; reference.

Model 3: Adjusted for age, sex, body mass index, marital status, equivalized income, regular exercise, history of hypertension, history of hyperlipidemia, history of diabetes mellitus, chronic pain, Pain Catastrophizing Scale score.

Model 4: Adjusted for age, sex, body mass index, marital status, equivalized income, regular exercise, history of hypertension, history of hyperlipidemia, history of diabetes mellitus, chronic pain, Pain Catastrophizing Scale score, history of depression, history of schizophrenia, and history of other mental diseases.

n = 1,942.

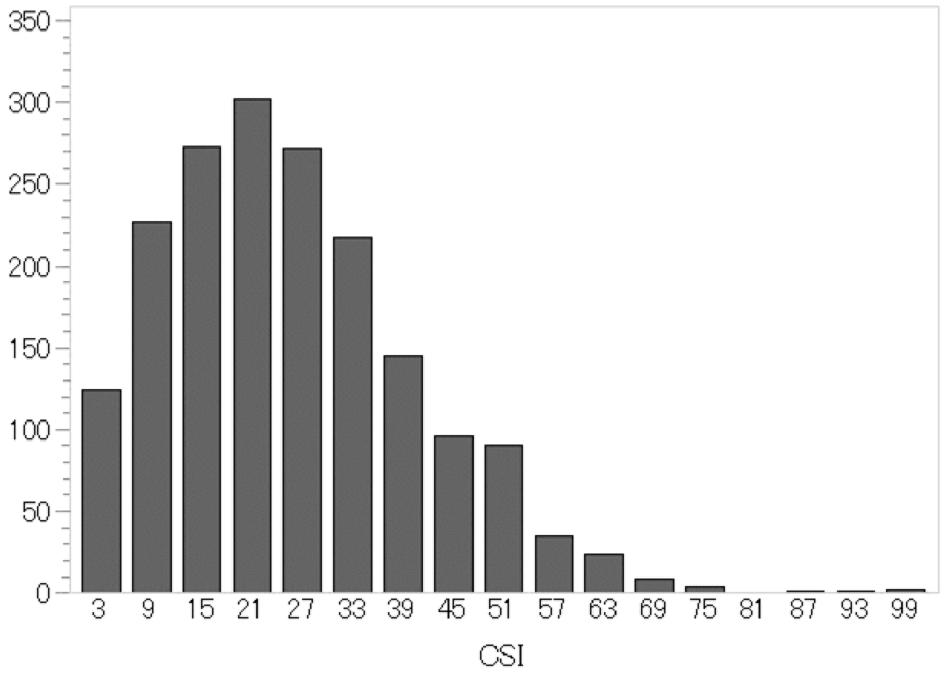

Figure S1. Distribution of central sensitization index (CSI) score
